# Next-Generation Sequencing Targeted Panel in Routine Care for Metastatic Colon Cancers

**DOI:** 10.3390/cancers13225750

**Published:** 2021-11-17

**Authors:** Arnaud Bayle, Debora Basile, Simon Garinet, Bastien Rance, Pierre Laurent-Puig, Hélène Blons, Julien Taieb, Geraldine Perkins

**Affiliations:** 1 Institut du Cancer Paris CARPEM, AP-HP, AP-HP.Centre, Department of Hepatogastroenterology and Digestive Oncology, Hôpital Européen Georges Pompidou, 20 Rue Leblanc, 75015 Paris, France; arnaud.bayle@aphp.fr (A.B.); debora.basile@aphp.fr (D.B.); geraldine.perkins@aphp.fr (G.P.); 2 Institut du Cancer Paris CARPEM, AP-HP, AP-HP. Centre, Department of Biochemistry, Hôpital Européen Georges Pompidou, 75015 Paris, France; simon.garinet@aphp.fr (S.G.); pierre.laurent-puig@aphp.fr (P.L.-P.); helene.blons@aphp.fr (H.B.); 3 Centre de Recherche des Cordeliers, INSERM, Sorbonne Université, Université de Paris, 75015 Paris, France; 4 Institut du Cancer Paris CARPEM, AP-HP, AP-HP.Centre, Department of Medical Bioinformatics, Hopital Européen Georges Pompidou, 75015 Paris, France; bastien.rance@aphp.fr

**Keywords:** next-generation sequencing, digestive cancer, colon cancer, oncology, genomics

## Abstract

**Simple Summary:**

The place of Next-Generation-Sequencing (NGS) targeted panel in routine practice in digestive oncology should be addressed. The aim of our retrospective study was to assess the results and impact of NGS panel for metastatic colorectal cancer (mCRC) patients. In total, 210 patients with mCRC were included. Based on our findings, a major advantage of the NGS panel over single gene techniques is that, beyond the classical hotspots, it allows for an exhaustive search for molecular abnormalities in routinely recommended genes. In addition, routine NGS is a way to detect amplifications associated with resistance to anti-*EGFR* therapies and low-prevalence mutations in actionable genes, providing patients with the opportunity to access innovative targeted therapies. In conclusion, NGS targeted panel in mCRC is feasible in routine practice. Nevertheless, panels need to be regularly updated and in-depth studies are needed to better analyse the prognostic factors.

**Abstract:**

In digestive oncology, the clinical impact of targeted next-generation sequencing (NGS) in routine practice should be addressed. In this work, we studied the impact of a 22-gene NGS amplicon-based panel with Ion Torrent Proton Sequencing, prospectively performed in routine practice. We analyzed the results of extended molecular testing, beyond *RAS* and *BRAF,* in metastatic colorectal cancer (mCRC) patients in a single-center, retrospective, observational study of consecutive mCRC patients followed up at the Georges Pompidou European Hospital between January 2016 and December 2018. Overall, 210 patients with mCRC were included. Median follow-up was 25.4 months (IQR: 14.9–39.5). The three most frequently mutated genes were: *TP53* (63%), *KRAS* (41%) and *PIK3CA* (19%). A positive association was found between overall survival and performance status (PS) ≥ 2 (HR: 4.91 (1.84–13.1); *p* = 0.001) and differentiation (HR: 4.70 (1.51–14.6); *p* = 0.007) in multivariate analysis. The NGS panel enabled five patients to access a targeted therapy not currently registered for CRC. In conclusion, targeted NGS panels in mCRC are feasible in routine practice, but need to be regularly updated and in-depth studies are needed to better analyze the prognostic factors.

## 1. Introduction

Next-generation sequencing (NGS) has revolutionized molecular tumor testing in oncology by offering the simultaneous assessment of many gene regions using formalin-fixed paraffin-embedded clinical samples. Sequencing technologies moved to high-throughput automated methods in the 2000s by focusing either on specific genes of interest (namely, targeted gene panels adapted to the disease), protein-coding sequences of DNA (whole-exome sequencing (WES)) or the entire genome (whole-genome sequencing (WGS)) and now allow for faster and easier sequencing coverage than ever before.

This has changed our capacity to screen tumor genomes and implement the findings in genetic diagnostics. However, given the ever-increasing technological advances and the different types of techniques (e.g., amplicon-based vs. hybridization capture-based) [1,2,3,4], there are new technical challenges in terms of the harmonization and quality assurance of NGS diagnostics and the interpretation of molecular changes and clinical relevance. 

Targeted panels focusing on specific genes of interest and adapted to the type of disease have been implemented in oncology. Due to the wide variety of drug targets in lung cancer, targeted NGS panels are nowadays widely used in routine practice, given their affordable cost and quick turnaround time. However, the impact and utility of multigene sequencing in daily practice in digestive cancer remain poorly described.

Among digestive cancers, colorectal cancer (CRC) is a major public health issue given its high incidence and mortality [5]. The recent improvement in overall survival is linked to an improved continuum of care through sequential treatment lines considering the molecular profiles of diseases. From the discovery of molecular biomarkers such as *KRAS* and *NRAS* mutations, which have been known to confer resistance to anti-*EGFR* therapy since the 2000s [6,7,8,9,10,11], over the last decade, we have moved to different molecular subtypes of metastatic CRC (mCRC) with different treatment options [12]. Therefore, molecular profiling has become essential and the guidelines recommend that all patients with mCRC should type tumor tissue for *RAS* (*KRAS* and *NRAS*) and *BRAF* mutations [13,14], and microsatellite instability (MSI) or mismatch repair (MMR) testing, as there are specific treatment options for wild-type *RAS*, *BRAF* V600E mutant and MSI mCRC [15]. 

To comply with the guidelines and recommendations and to improve therapeutic options in the future, a more comprehensive molecular characterization is needed, and academic centers have implemented multigene sequencing with new prognostic or predictive biomarkers to individualize treatment decisions and widen treatment options with, for example, innovative targeted therapies. Some previous studies have highlighted that NGS is feasible in CRC, as it uses a limited amount of tissue [16], is affordable cost, and can be conducted in a timely manner. However, these studies were only conducted in small CRC cohorts with mostly localized cancers [17], or in the context of clinical trials [18]. Moreover, despite more recent studies [19,20], clinical practice guidelines and recommendations for the use of NGS in this setting have not changed [21]. 

In our center, NGS panels were implemented from 2016, in routine diagnostics, independently of the type of tumor, alongside mutation-specific TaqMan assays for the rapid identification of mutations in a subset of frequent alterations. For patients with digestive cancers, and particularly patients with mCRC, NGS offers a potential benefit, as it allows for the detection of rare *RAS*/*BRAF* mutations or molecular alterations that could impede the anti-*EGFR* response, the identification of potential drug targets (*HER2*, *PIK3CA*.), and the possibility of testing the clinical value of co-occurring alterations. 

This study reports the results of mCRC molecular testing using NGS as a routine diagnostic tool and discusses the benefit of testing many genes at once, and thereby yielding more molecular diagnoses for patients with digestive cancers.

## 2. Materials and Methods

### 2.1. Methodology Overview

Tumor testing was performed following standard laboratory procedures for molecular analysis, including selection of tumor block, assessment of tumor cell percentage, DNA extraction and quantification and molecular testing. Molecular analyses are performed in 2 steps: rapid screening of frequent mutations in *KRAS* and *BRAF* and subsequent NGS using a 22-gene panel. Analyses were prospectively performed in routine practice for digestive cancers in our institution (Georges Pompidou European Hospital, Paris, France) between January 2016 and December 2018. A retrospective analysis of the clinical data was carried out and all patients who fulfilled the inclusion criteria were selected for analysis. The study was approved by the Ethics Committee of Georges Pompidou European Hospital (Ref. 2020-11-04).

### 2.2. Inclusion Criteria

Patients who met all the following criteria were included: Patients > 18 years old with pathologically proven metastatic CRC, with a least one NGS performed at our hospital in routine practice between 2016 and 2018, on either a primary tumor or metastatic tissue. 

### 2.3. Exclusion Criteria

Patients with any one of the following issues were excluded: patients with exclusively localized, benign or in situ tumors, or with any other ongoing malignancy, or followed up exclusively in another hospital, or without any conclusive NGS result, or NGS only performed on a liquid biopsy. 

### 2.4. Patient Characteristics

All clinical, histological, radiological and laboratory parameters were retrospectively collected. Clinical data included the number of lines of treatment received by patients and whether they received anti-*EGFR* or anti-*VEGF* therapy. Histological parameters included differentiation (poorly differentiated/undifferentiated vs. well or moderately differentiated) and TNM stage. Radiological data included evaluation of the disease according to the RECIST criteria 1.1. Laboratory data included CEA and CA 19.9 levels measured at initiation of first-line treatment of metastatic disease.

### 2.5. TaqMan Probe Testing for KRAS and BRAF

For single-gene assays, allele-specific qPCR technology was performed using TaqMan probes for *KRAS* (p.Gly12Ser/Cys/Asp/Ala/Val and p.Gly13Asp) and for *BRAF* p.Val600Glu (Thermo Fisher Scientific, in-house design, available on request, Waltham, MA, USA).

Each real-time quantitative PCR, one per probe, was run in a final volume of 5 µL in 384-well plates, including 2.5 µL of 2× TaqMan genotyping master mix (Applied Biosystems, Foster City, CA, USA), 0.5 µL of 10× Assay Mix, 1 µL of deionized water, and 10 ng of DNA template. Samples were run in duplicate on an ABI Prism 7900 HT sequence detection system (Applied Biosystems) using standard thermocycling conditions and analyzed with SDS software version 2.4 (Applied Biosystems). *KRAS* and *BRAF* mutation status was determined according to the manufacturer’s instructions and the results obtained for these 2 genes, in the hotspots defined above, were combined, and compared with those obtained in NGS to define the mutation status of the patients.

### 2.6. Multigene Sequencing by NGS 

Independently of the mutational status obtained using probes, NGS was performed with a dedicated panel of 92 amplicons (Ion AmpliSeq Colon-Lung Cancer Research Panel version 2; Life Technologies, Carlsbad, CA, USA), covering > 500 hotspot mutations in *KRAS*, *EGFR*, *BRAF*, *PIK3CA*, *AKT1*, *ERBB2*, *PTEN*, *NRAS*, *STK11*, *MAP2K1*, *ALK*, *DDR2*, *CTNNB1*, *MET*, *TP53*, *SMAD4*, *FBXW7*, *FGFR3*, *NOTCH1*, *ERBB4*, *FGFR1*, and *FGFR2*. Multiplex PCR libraries were prepared using 30 ng of DNA whenever possible and 3 µL of DNA for samples with DNA concentration < 10 ng/µL by AmpliSeq technology (Ion AmpliSeq library kit version 2, Ion library equalizer kit; Life Technologies). Clonal amplification and sequencing were done on the Ion Chef System (Ion PI Hi-Q Chef, Ion PI Chip Kit v3) and Ion Torrent Proton sequencer (Life Technologies).

Data were analyzed by the Torrent Suite 4.4.3 and 5.0.4 (Life Technologies) using optimized parameters: minimal depth 300×, detection threshold of 2% and 1% for hotspots. Variant call files from the variant caller were loaded on a galaxy platform and annotated using the Safir2report tool (W Digan et al. GigaScience, Volume 6, Issue 11, November 2017, gix099, https://doi.org/10.1093/gigascience/gix099, accessed on 5 September 2021). NGS coverage depth data were used to identify gene amplifications (in particular *ERBB2*) using an algorithm developed in our laboratory (Legras et al. Suplementary data [22]) based on the identification of outliers from the expected coverage mean + 3SD and calculated using all of the run data. 

Mutated genes were classified in different groups to facilitate NGS interpretation:

- **NGS mutations with validated clinical impact**: *KRAS*, *BRAF*, *NRAS;*
- **NGS mutations with potential clinical impact**: *PIK3CA*, *AKT1*, *ERBB2*, *PTEN*, *STK11*, *MAP2K1*, *ALK*, *MET*, *FGFR1*, *FGFR2*, *FGFR3*, *ERBB4*, *EGFR*;
- **NGS mutations with unknown clinical impact**: *DDR2*, *CTNNB1*, *TP53*, *SMAD4*, *FBXW7*, *NOTCH1.*

### 2.7. MSI Status 

#### 2.7.1. Immunohistochemistry 

For mismatch repair protein (MMRP) status, immunohistochemistry (IHC) was performed by pathologist on a tumor sample using a four-antibody panel including MLH1, MSH2, MSH6, and PMS2.

#### 2.7.2. Molecular Test

MSI status was analyzed using the MSI kit (Promega, France). The analysis of five mononucleotide microsatellites (BAT-25, BAT-26, NR-21, NR-24 and MON-27) is recommended by the revised Bethesda guidelines [23] and the ESMO guidelines [24] for mCRC MMR status determination. Tumors were defined as high-frequency microsatellite instability (MSI-H) when two or more of the five markers in the tumor DNA were positive. If none or only one of the markers showed instability, the tumor was considered to be microsatellite stable (MSS).

### 2.8. Statistical Analysis

The data were analyzed with Fisher’s exact test, or the Chi-square test was used for categorical variables as appropriate. The impact of clinicopathological factors and gene mutations on progression-free survival (PFS) under biotherapy (anti-*EGFR* or anti-*VEGF*) and on overall survival (OS) was analyzed using Kaplan–Meier curves with the log-rank test. Multivariate COX analysis was employed using stepwise regression (forward: LR), and all factors with statistical significance in univariate analysis were included in multivariate analysis. *p* < 0.05 (two-sided) was considered as statistically significant. Study data were collected and managed using REDCap electronic data-capture tools hosted at Georges Pompidou European Hospital. REDCap (Research Electronic Data Capture) [25,26] is a secure, web-based software platform designed to support data capture for research studies. 

## 3. Results

### 3.1. Patients

**Figure 1:**
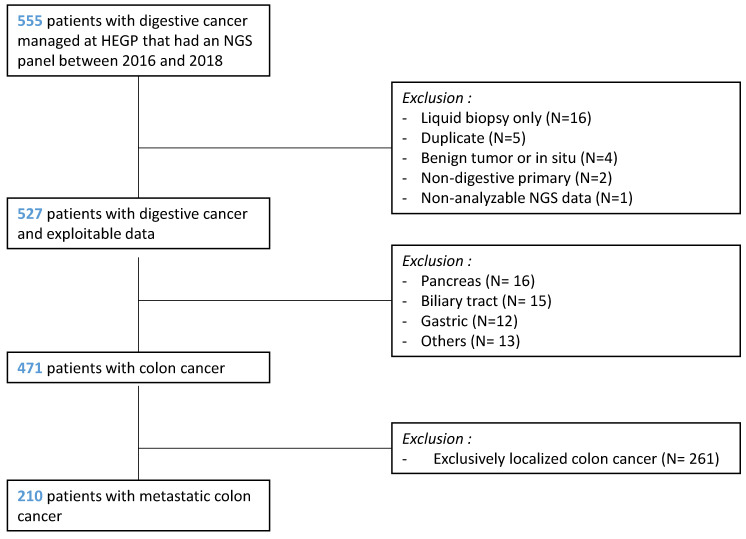
Flowchart.

Between January 2016 and December 2018, an NGS targeted panel analysis was performed for 555 patients managed for a digestive cancer at Georges Pompidou European Hospital (HEGP). After excluding patients with no exploitable data (*N* = 28) and patients with a cancer other than CRC (*N* = 56), 471 patients with CRC were identified and 210 patients with metastatic colon cancer were finally included (Figure 1). 

Median follow-up was 25.4 months (IQR interquartile range: 14.9–39.5). Median age was 67.5 years (IQR: 58.1–76.2) with a majority of patients (*N* = 168, 80%) in good general condition, with PS 0 or 1 at diagnosis. Of the 210 metastatic patients, 140 (67%) were metastatic at diagnosis and 186 (89%) patients received at least one line of systemic therapy (Table 1). 

### 3.2. Mutational Profile

For the mCRC population, mutations found with the NGS panel are summarized in Table 2. The 3 most frequently mutated genes were: *TP53* (*n* = 132, 63%), *KRAS* (*n* = 86, 41%) and *PIK3CA* (*n* = 39, 19%); a *BRAF* mutation was found in 20 patients (10%). TaqMan mutation testing identified a *KRAS* mutation in only 68 (vs. 86 with NGS) patients and a *BRAF* mutation in only 15 patients (vs. 20 with NGS). 

Concerning the number of variants per patient found by NGS, 16 (8%) patients had no mutation in the genes studied. Among the patients with at least one variant, 67 (34%) had 1 variant, 72 (36%) 2 variants, 29 (15%) 3 variants and 15 (7%) 4 or more variants. Amplifications were found in *ERBB2* (*n* = 5, 2.5%), *FGFR3* (*n* = 4, 2%), *MET* (*n* = 3, 1.5%) and *KRAS* (*n* = 3, 1.5%). 

Of the 183 patients screened for MMR deficiency by immunohistochemistry, 19 (10%) had a tumor with a dMMR phenotype. Of the 165 patients screened for MMR deficiency in molecular biology, 19 (12%) had an MSI tumor. Combining the results obtained by the two techniques, we found 22 (11%) patients with an MMR deficiency defined either by immunohistochemistry or molecular biology. MMR deficiency was statistically more frequently associated with a *BRAF* mutation (41% vs. 6.8%, *p* < 0.001), a right tumor location (77% vs. 33%, *p* < 0.001), a higher median age (77.6 years vs. 66.4 years, *p* = 0.01), a higher female-to-male ratio (68% vs. 45%, *p* = 0.06), *PTEN* mutations (18% vs. 1.2%, *p* = 0.002) and a high number of variants in NGS (number of variants ≥2 in 45% of MMR-deficient patients vs. 20% of MMR-proficient patients, *p* = 0.015).

### 3.3. Treatments Characteristics

Of the 210 patients with mCRC included in our study, 176 received systemic treatment (84%). Of these, 64 (36%) received 1 line of treatment, 48 (27%) 2 lines, 33 (19%) 3 lines, and 31 (18%) 4 lines or more. Among these patients, 53 (52%) of them received first- or second-line anti-*EGFR* therapy (*n* = 45, 85%). The PFS under anti-*EGFR* treatment was 8.1 months (IQR: 4.40–16.20), and 32 (63%) patients exhibited an objective response. 

In addition, 98 (46.7%) metastatic patients received first- and/or second-line antiangiogenic therapy, 83 (86%) of them. Median PFS on antiangiogenic therapy was 7.33 months (IQR: 4.26–13.8) and 31 (33%) patients had an objective response. 

Finally, 5 patients with actionable alterations detected by NGS had access to a targeted therapy. Two patients received an anti-*HER2* inhibitor, 1 patient an anti-*FGFR* inhibitor, 1 patient anti-*MET*/anti-*MEK* therapy and 1 patient anti-*ALK* therapy. Among these patients, 2 (40%) received the treatment in the context of a clinical trial and 3 (60%) as off-label, after validation by the Georges Pompidou European Hospital molecular tumor board. As best responses, 1 partial response, 2 tumor stabilizations and 1 immediate progression (1 patient was not evaluable) were observed (Supplementary Table S1).

### 3.4. Univariate Analysis 

In univariate analysis, there was a statistically significant negative association between *BRAF* gene mutation and OS in Taqman (HR = 0.41 (0.20–0.83); *p* = 0.013 and NGS (HR = 0.43 (0.23–0.83); *p* = 0.012). Of note, there was also a statistically significant negative association between mutation in NGS of the *FGFR3* gene (HR = 0.03 (0.00–0.26); *p* = 0.001) and the *MAP2K1* gene (HR = 0.03 (0.00–0.26); *p* = 0.001) and OS; however, given the small number of patients with a tumor harbouring these mutations, these results were not included in the multivariate analysis. Moreover, there was no statistically significant negative association between the number of variants in NGS and OS, even for 4 or more variants (HR = 1.40 (0.60–3.23); *p* = 0.4). However, there was a clear trend towards significance between OS and MSI status in IHC (HR= 2.11 (1.00–4.45), *p* = 0.05).

In univariate analysis, there was a negative association between OS and the following clinical criteria: PS ≥ 2 (HR: 3.67 (2.10–6.42), *p* < 0.001), T4 primary tumor (HR: 1.90 (1.05–3.44), *p* < 0.034) and poorly differentiated adenocarcinoma (HR: 2.82 (1.25–6.35), *p* = 0.012) (Table 3). 

### 3.5. Multivariate Analysis

For the multivariate analysis of OS, we selected all variables that were statistically significant in the univariate analyses and only a PS ≥ 2 (with, respectively, HR: 4.91 (1.84–13.1) *p* = 0.001) and a poorly differentiated adenocarcinoma (HR: 4.70 (1.51–14.6)) *p* = 0.007) remained associated with a poorer OS (Table 3).

### 3.6. Cost and Turnaround Time

#### 3.6.1. TaqMan 

The commercial cost of reagents for PCR with the use of TaqMan probes has already been described in a previous study [27] and ranged from 5.5 € to 19.0 €. With 2 rounds of analysis organized per week, the turnaround time for the result was about 72 h from receipt of the tissue.

#### 3.6.2. NGS

The commercial cost of the NGS panel reagents used in this study has already been described in a previous study [22] and amounts to 82 €. With one session per week on average, the turnaround time for the result ranged from 7 to 14 days from receipt of the tissue. 

## 4. Discussion

To our knowledge, there are no real-world studies looking at the impact of NGS on a large series of patients with mCRC. One of the major interests of this study is that we examined the clinical value of targeted sequencing in a representative series of CRC patients encountered in day-to-day care oncology units. Our study shows that, in parallel with single-gene assays, targeted NGS is feasible in routine practice at an affordable cost, and enables better tumor characterization. Based on our findings, a major benefit of NGS panels over single-gene techniques is that, beyond the classical hotspots, it allows for an exhaustive search for molecular abnormalities in routinely recommended genes. NGS enabled the identification of 18 additional samples with rare *KRAS* alterations (68 patients identified with *KRAS* mutation with Taqman vs. 86 with NGS) and 10 with *NRAS.* All additional *RAS* mutations identified by NGS were included in Exon2, 3 and 4, and therefore involved in the anti-*EGFR* therapy decision. Therefore, a comprehensive *RAS* analysis may prevent the use of *EGFR* inhibitors for patients who are not expected to benefit from this treatment. NGS further enabled the identification of five additional patients with *BRAF* mutation (15 patients identified with *BRAF* with Taqman mutation vs. 20 with NGS; of note, the additional *BRAF* mutations identified by NGS were non-V600E mutations, but the evidence shows that they are associated with some therapeutic results [28,29]). Moreover, the identification of alterations related to resistance to *EGFR* treatment such as *HER2* mutations (*n* = 2) or amplification (*n* = 5), *FGFR* mutations (*n* = 5), and *KRAS* amplification (*n* = 3) may also help select the best treatment for patients with mCRC.

Indeed, in addition to the rare mutations detected by NGS, these panels also make it possible to detect amplifications, which, for instance, in the case of *KRAS* or *MET*, are associated with resistance to anti-*EGFR* therapy and are, therefore, also an important part of the therapeutic decision. Although the speed of execution of single-gene techniques remains one of their main justifications, NGS panels take only slightly longer and use less tissue for a larger number of genes of wider scope. 

Moreover, given the fact that some potential targetable alterations have low prevalence and are outside those recommended in routine practice, NGS enables the identification of patients with these alterations. Therefore, NGS is a way of ensuring that patients have access to innovation, for instance, through clinical trials in which drugs targeting these alterations are being developed. In our experience, few patients (2.4%) have benefited from targeted therapy, but this can be explained by the our study’s timeframe, which does not consider recent developments and trials that have tested many new innovative targeted therapies within the last 2–3 years. Indeed, the current data show that patients with MSI-H tumors can benefit from immunotherapy [30], while patients with a *BRAF* V600E mutation can benefit from encorafenib and cetuximab beyond first-line treatment [31], and non-randomized phase I and II trials have raised new hopes for mCRC with *ERBB2* alterations or *KRAS* G12C mutations [32,33,34,35]. Of note, in our series, *ERBB2* amplifications were found in 2.5% patients which is consistent with data in the literature, such as the AACR GENIE database [36]. 

**Figure 2:**
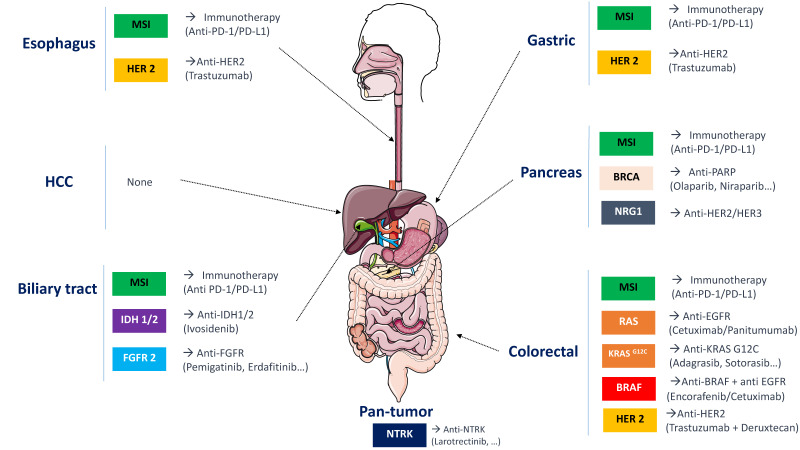
Major molecular abnormalities in digestive cancers and current treatment options.

As illustrated by Figure 2, this is a global trend which, beyond colon cancer, concerns all gastrointestinal cancers. Therefore, we can expect that the therapeutic options following an NGS result will be wider in future, with the arrival of new drugs. 

Another study, published in 2018 by Gao et al. [17], investigated the predictive and prognostic factors of an identical NGS panel in 207 patients with CRC. Unlike this publication, we have not identified a link between the total number of variants and OS. However, it is important to underline that this study was carried out on a different population of Chinese patients, who mainly had localized colon cancer, and NGS was performed only on primary tumor tissue and in a very different molecular landscape (e.g., 59% with *TP53* mutation in our study vs. 74% in Gao et al.). In addition to the potentially greater confounding factors, this difference may also be explained by a lack of power, with an insufficient number of patients with low-prevalence mutations. Similarly, the association between overall survival and MSI status, although significant, remains weak in our series, possibly due to sampling fluctuations.

Our study has limitations that deserve to be mentioned. First, the targeted NGS panel is not specific for CRC tumors, was restricted to 22 genes and does not detect gene fusions. With new drug developments, broader DNA/RNA panels should be validated, allowing for a comprehensive screening of actionable alterations. Recently, new drugs such as larotrectinib, have been approved by the FDA for tumors (including rare cases of CRC) with *NTRK* fusions. *POLE* hotspot mutations could predict sensitivity to immune checkpoint inhibitors.

Second, unlike clinical trials such as MOSCATO [37] and SHIVA [38], the treatment decision was not based on the NGS results. Indeed, in daily practice, as the routine use of NGS was not the standard, it had no impact on the recommended standard of care for the first lines of treatment, except for rare *RAS* mutations that were not detected by the TaqMan method. Therefore, NGS data were only considered later during cancer progression after standard management, so as to eventually propose enrolment in a clinical trial or use of an off-label therapy for fit patients still willing to be treated. Unfortunately, many of these heavily pre-treated patients were not fit enough to receive a new line of treatment. This may also explain the low number of cases of patients with matched therapy who derived potential benefits from the results of molecular analysis by NGS, but this reflects the clinical reality.

Third, we failed to identify genomic prognostic factors with this NGS panel. The first possible reason for this is that molecular analysis was performed either on the primary tumor or on the metastasis and changes may have occurred during progression. The second possible reason is the molecular and clinical heterogeneity of the patients. Tumors have different molecular profiles and different co-alterations and the study population was not large enough to test all subgroups. Patients also have different disease histories: some were naïve to any treatment and some were heavily pre-treated. It would be interesting to conduct more in-depth studies on each of these clinical situations, with more patients, to better analyze the predictive and prognostic factors that could be discovered by an accurate NGS panel. 

In addition, our NGS panel does not allow for identification of the consensus molecular subtypes [39], which distinguishes four molecular subtypes in 80% of CRCs, or the tumor mutational burden [40], which may suggest the use of immunotherapy in MSS mCRC patients. However, the classification of consensus molecular subtypes is not routinely used because it requires the analysis of more than 80 genes, as does the calculation of tumor mutational burden, which requires the analysis of several hundred genes to be relevant. 

Nevertheless, routine NGS screening has several advantages. First, it completely screens validated molecular predictors (*RAS*) or alterations related to *EGFR* inhibitor sensitivity (*RAS* amplification, *HER2*). Thus, it identifies potential targets such as *BRAF* or *HER2*. Finally, it is robust enough to work on low-quality FFPE DNA and has a high sensitivity in the detection of variants in small tumor cell samples. However, it is important to note that it does not seem to have prognostic value in an unselected series of patients. Indeed, here, we show that clinical criteria (e.g., general condition) or pathological criteria (e.g., tumor differentiation) remain very important and are mandatory for diagnosis and treatment decision making. However, broad molecular profiling can guide treatment choices and open therapeutic options. In the era of precision medicine, integrating high-throughput sequencing into the patient care process appears to be central to offering patients innovative targeted therapies directed against the specific molecular anomalies. Therapeutic decisions have to be evaluated using molecular tumor boards, especially for off-label treatment use, when patients are not eligible for industrial or academic trials.

## 5. Conclusions

This study shows that, in parallel with single-gene assays, NGS-targeted panels in metastatic colon cancer are feasible in routine practice at an affordable cost, and improve tumor characterization. However, these panels need to regularly be updated to ensure that they remain relevant and increase the number of patients who can directly benefit from them.

## Figures and Tables

**Table 1:** Patient characteristics.

Variable (*N*=)		Median (IQR) (%)	
Number of Patients		210	
Median follow-up (months)		25.4	(14.9–39.5)
Sex	Female	101	(48)
	Male	109	(52)
Age		67.5	(58.1–76.2)
Location (*N* = 207)	Right Colon	72	(35)
	Left colon	102	(49)
	Rectum	33	(16)
Metastasis	Synchronous	140	(67)
	Metachronous	70	(33)
Systemic treatment	Yes	186	(89)
(*N* = 207)	No	24	(11)
Performance status (at first-line treatment of metastatic disease)	0	75	(37)
(*N* = 203)	1	93	(46)
	2	31	(15)
	3	4	(2)
CEA ^1^ (*N* = 181)		9.5	(3–45)
CA 19.9 ^1^ (*N* = 170)		39	(13–246)
Specific targeted therapy based on NGS results	Clinical trial	2	(40)
	Off-label	3	(60)

^1^ Measured at initiation of first-line treatment of metastatic disease.

**Table 2:** Molecular alterations.

Variable		Median (Range)/*N* (%)		
Molecular alteration on NGS panel (*N* = 199)		*N* (%)		
Gene mutations	*TP53*	132 (63)	*ERBB4*	4 (2)
	*KRAS*	86 (41)	*ERBB2*	2 (1)
	*PIK3CA*	39 (19)	*FGFR2*	2 (1)
	*BRAF*	20 (10)	*NOTCH1*	2 (1)
	*SMAD4*	20 (10)	*DDR2*	2 (1)
	*FBXW7*	12 (6)	*MAP2K1*	2 (1)
	*NRAS*	10 (5)	*ALK*	1 (0.5)
	*PTEN*	7 (3)	*FGFR1*	1 (0.5)
	*AKT1*	5 (2)	*STK11*	1 (0.5)
	*CTNNB1*	4 (2)	*FGFR3*	1 (0.5)
Amplifications	*ERBB2*	5 (2.5)	*MAP2K1*	2(1)
	*FGFR 3*	4 (2)	*NOTCH 1*	1 (0.5)
	*MET*	3 (1.5)	*PIK3CA*	1 (0.5)
	*KRAS*	3 (1.5)	*FGFR2*	1(0.5)
	*BRAF*	2(1)		
Number of variants/patient	0	16 (8)		
	1	67 (34)		
	2	72 (36)		
	3	29 (15)		
	4 et +	15 (7)		
Microsatellite instability status				
Immunohisto-chemistry	MMR proficiency	164 (90)		
(*N* = 183)	MMR deficiency	19 (10)		
Molecular biology	MSS	146 (88)		
(*N* = 165)	MSI	19 (12)		
	Not evaluable	4 (3)		
Molecular alteration (TaqMan) (*N* = 196)				
*KRAS*	WT	128 (65)		
	Mutated	68 (35)		
*BRAF*	WT	181 (92)		
	Mutated	15 (8)		

**Table 3:** Univariate and multivariate analysis on overall survival (OS).

Variable	OS Hazard Ratio [95% CI] *p*-Value	
	Univariate Analysis	Multivariate Analysis
Performance status (≥2 or <2)	3.67 (2.10–6.42) <0.001	4.91 (1.84–13.1) 0.001
Differentiation (poorly differentiated/undifferentiated vs. well or moderately differentiated)	2.82 (1.25–6.35) 0.012	4.70 (1.51–14.6) 0.007
T (T4 vs. T1/T2/T3)	1.90 (1.05–3.44) 0.034	0.95 (0.51–2.63) 0.73
Microsatellite instability status	0.47 (0.22–1) 0.05	1.05 (0.23–4.70) 0.9
*BRAF* (mutated vs. wild-type)	2.44 (1.21–4.93) 0.013	1.75 (0.46–6.58) 0.41

## Data Availability

The data that support the findings of this study are available from the corresponding author upon reasonable request.

## Supplementary Materials

The following are available online at https://www.mdpi.com/article/10.3390/cancers13225750/s1. Table S1: Treatment characteristics.
