# Supplementary material for: Next-Generation Sequencing Targeted Panel in Routine Care for Metastatic Colon Cancers
Source: Cancers (Basel). 2021 Nov 17;13(22):5750. doi: 10.3390/cancers13225750 (PMC8616114; doi:10.3390/cancers13225750)
Supplement: Supplementary file 1 [file cancers-13-05750-s001.zip › cancers-1433094-supplementary.pdf]

Supplementary

Table S1: Treatment characteristics

Variable		Median (IQR)/N (%)	
**Anti-EGFR treatment**	Yes	53	(25)
	No	157	(75)
**- Line of first anti-EGFR treatment** (N=53)	1	30	(56.6)
	2	15	(28.3)
	3	6	(11.3)
	> or = 4	2	(3.8)
**- PFS on anti-EGFR treatment (months)**		8.08	(4.40-16.20)
**- Best response on anti-EGFR treatment** (N=52)	CR	0	(0)
	PR	32	(63)
	SD	15	(29)
	PD	4	(8)
**Anti-VEGF treatment** (N=210)	Yes	98	(46.7)
	No	112	(53.3)
**- Line of first anti-VEGF treatment** (N= 97)	1	56	(58)
	2	27	(28)
	3	11	(11)
	> or = 4	3	(3)
**- PFS on anti-VEGF treatment (months)**		7.33	(4.26-13.8)
**- Best response on anti-VEGF treatment?** (N= 94)	CR	2	(2)
	PR	29	(31)
	SD	50	(53)
	PD	13	(14)
**Type of targeted therapy post-NGS**	Anti-HER2	2	(40)
	Anti-FGFR	1	(20)
	Anti-ALK	1	(20)
	Anti-MET/Anti-MEK	1	(20)
**Mechanism of access to targeted therapy**	Clinical Trial	2	(40)
	Off-label	3	(60)
